# Characteristics of heart failure complicated by dementia

**DOI:** 10.1002/alz.087797

**Published:** 2025-01-09

**Authors:** Manabu Kokubo, Atsuya Shimizu, Takahiro Kamihara, Akihiro Hirashiki, Akinori Takeda, Hidenori Arai

**Affiliations:** ^1^ The National Center for Geriatrics and Gerontology, Obu, Aichi Japan; ^2^ National Center for Geriatrics and Gerontology, Obu, Aichi Japan

## Abstract

**Background:**

With its rapidly aging population, Japan has been seeing an increase in the concurrent incidence of dementia during heart failure treatment, making disease management difficult. Nevertheless, not much has been uncovered about the actual state of the treatment status and prognosis of heart failure patients with dementia.

**Method:**

At the National Center for Geriatrics and Gerontology, we retrospectively analyzed cases of patients who died of heart failure between 2018 and 2022, who were also complicated with dementia, and whose treatment progress from the initial onset of heart failure to death could be confirmed.

**Result:**

Overall, 20 patients satisfied the above criteria. Their mean age at death was 89.4 ± 3.3 years (10 males: 89.3 ± 2.3 years; 10 females: 89.5 ± 3.7 years). The mean duration from the initial onset of heart failure (heart failure stage C) to death was 2.4 ± 3.3 years, with a maximum of approximately 11 years and a minimum of several days.

The mean number of hospitalizations during the course of the disease was 2.1 ± 1.4, with the highest number being 6, and 9 patients ending up dying during the first hospitalization. Four patients developed dementia after the onset of heart failure, and their treatment duration tended to be prolonged. Only four patients were institutionalized at the time of the onset of heart failure. The most common underlying disease was hypertension, which was observed in 19 patients. Additionally, atrial fibrillation, chronic kidney disease, valvular heart disease, and ischemic heart disease were noted in 9, 10, 6, and 6 patients, respectively. Regarding the classification of heart failure, 12 patients had HFpEF, and 8 had HFrEF

**Conclusion:**

The 5‐year survival rate following the initial onset of heart failure is reported to be 75%. However, a poorer prognosis is likely expected if a heart failure patient is complicated with dementia. The possible factors behind this include the onset of the disease upon entering the older age, the status of oral medications, and difficulty in self‐management. A more detailed examination of the actual situation will be required.

